# Effect of Lifestyle Interventions during Pregnancy on Maternal Leptin, Resistin and Offspring Weight at Birth and One Year of Life

**DOI:** 10.3390/biomedicines11020447

**Published:** 2023-02-03

**Authors:** Nina Ferrari, Nikola Schmidt, Lisa Schmidt, Waltraut M. Merz, Konrad Brockmeier, Jörg Dötsch, Inga Bae-Gartz, Esther Mahabir, Christine Joisten

**Affiliations:** 1 Cologne Center for Prevention in Childhood, Youth/Heart Center Cologne, University Hospital of Cologne, Kerpener Str. 62, 50937 Cologne, Germany; 2 Department for Physical Activity in Public Health, Institute of Movement and Neurosciences, German Sport University Cologne, Am Sportpark Müngersdorf 6, 50933 Cologne, Germany; 3 Department for Pediatric Cardiology, Heart Center, University of Cologne, Kerpener Str. 62, 50937 Cologne, Germany; 4 Department of Obstetrics and Prenatal Medicine, Venusberg-Campus 1, University Bonn Medical School, 53127 Bonn, Germany; 5 Department of Pediatrics and Adolescent Medicine, Faculty of Medicine, University of Cologne, Robert-Koch-Str. 16, 50931 Cologne, Germany; 6 Comparative Medicine, Center for Molecular Medicine, University of Cologne, Faculty of Medicine and University Hospital of Cologne, Robert-Koch-Str. 21, 50931 Cologne, Germany

**Keywords:** physical activity, leptin, resistin, fat mass, foetal outcome

## Abstract

Lifestyle during pregnancy impacts the health of the mother and child. However, the extent to which physical activity affects maternal biomarkers and factors that might influence birth weight remains unclear. We analysed data from two lifestyle interventions in which the effects of an exercise programme (2x/week, 60–90 min) on the course of pregnancy with regard to adipokines and offspring were evaluated. A total of 70 women participated in this study (45, intervention group; 25, control group). Anthropometric data and maternal fasting serum leptin and resistin levels were measured at three time points (approximately 14th (T1), 24th (T2), and 36th (T3) weeks of gestation). Neonatal/child data were retrieved from screening examinations. Independent of the intervention, we found a positive correlation between the fat mass at T1 and both leptin and resistin levels at all time points. Leptin level was significantly higher in the control group at T3; however, no differences between the groups were found for resistin. The birth weight was influenced by the birth length, fat mass at T1/T3, and resistin level at T2. The BMI-SDS at one year of age was influenced by maternal fat-free mass at T3 and resistin at T1/T2. Even if these results can only be interpreted cautiously, lifestyle interventions during pregnancy are important in promoting maternal and child health. Further randomised controlled trials and translational studies are warranted to clarify the underlying mechanisms.

## 1. Introduction

Lifestyle behaviours during pregnancy have a major impact on subsequent generations [1,2]. Maternal overweight, gestational diabetes, or excessive weight gain during pregnancy thus increases the risk of macrosomia in newborns. Infants with an increased birth weight are also at a significantly increased risk of becoming overweight and developing obesity later in life, accompanied by dysfunction in glucose metabolism and insulin secretion and sensitivity [3,4].

This can be explained by cellular and molecular biological factors, especially metabo-inflammatory effects and interactions between the mother and child [5,6]. In this context, the best-studied factor is insulin, which plays an important role in regulating energy and glucose homoeostasis at different levels inside and outside the hypothalamus [7]. Physiological changes during pregnancy include a decrease in insulin sensitivity in various organ systems, such as white adipose tissue or the liver [8]. These changes are notably more pronounced and are associated with a threefold higher risk for gestational diabetes mellitus (GDM) in pregnant women with overweight/obesity than in those with normal weight [9,10]. The development of GDM is supported by leptin [11], which affects centres in the hypothalamus that regulate food intake, body weight, and metabolism, such as the arcuate nucleus [12]. Studies have also shown that elevated leptin levels in the first and second trimesters of pregnancy are associated with the development of GDM [13]. Regarding birth weight, Garofoli et al. recently identified a negative correlation with maternal leptin levels upon delivery [14]. However, this correlation became insignificant after taking into account possible confounders, such as maternal body mass index (BMI). Less attention has been given to resistin, which is secreted from not only (visceral) adipose tissue but also the placenta [15] and seems to be associated with the development of insulin resistance and obesity. Resistin is a pro-inflammatory protein that is mainly synthesised by macrophages in humans [16] and is related to the expression of pro-inflammatory mediators such as tumour necrosis factor alpha (TNF-α) or interleukin 6 (IL-6).

In the context of pregnancy, studies have indicated elevated resistin levels in women with GDM and pre-eclampsia [17,18,19]. Furthermore, a positive correlation exists between maternal resistin levels and the HOMA-IR [20]. Whether maternal resistin levels also influence birth weight remains controversial. While Jansson et al. found a correlation between high first-trimester maternal serum resistin levels and increased birth weight, Vitoratos et al. did not identify any correlation between maternal resistin levels from the 24th to 26th week of gestation and birth weight [21,22]. Wang et al. also noted a positive correlation between resistin levels in umbilical cord blood and birth weight; umbilical resistin levels were significantly higher in children with a birth weight of >3500 g than in those with a birth weight of <3000 g [23]. In addition, in their study of 40 pregnant women and the respective newborns, Marinoni et al. showed that both foetal and maternal resistin levels at delivery were positively associated with birth weight [24].

The impact of lifestyle behaviours has been discussed mainly from the perspective of weight gain; therefore, most studies have focused on energy intake or dieting. However, body composition is also decisively influenced by physical activity and its effects on the muscles; investigations into the role of the so-called myokines or exerkines in the context of pregnancy are still in the early stages [25]. Previous studies have indicated that exercise during pregnancy can reduce leptin levels [15,26]. Ning et al. showed that the mean leptin levels were lower in women with the highest levels of physical activity (>12.8 h/week) and energy expenditure (>70.4 metabolic equivalent of task; MET h/week) than in inactive women during early pregnancy (average of 12–13 weeks of gestation) [26]. Van Poppel et al. found significantly reduced cord blood leptin levels in 436 pregnant women with obesity after a diet- and exercise-based intervention compared with those in controls [27].

Resistin levels are also reduced through physical activity in the non-pregnant state [28,29]. For example, in their cross-sectional study of 6636 adults (men and non-pregnant women), Marcelino-Rodriguez et al. showed that the levels of resistin were inversely correlated with the duration of leisure-time physical activity, leisure-time MET, and moderate leisure-time physical activity [30]. Women in the upper quantile of leisure-time physical activity were at a lower risk of displaying elevated resistin levels. In addition, the risk of elevated serum resistin levels was lower in individuals who spent more than 20 min/day on physical activity during their leisure time. To the best of our knowledge, no studies have yet investigated the effects of physical activity on resistin levels during pregnancy. Therefore, the present study aimed to investigate the impact of a multimodal intervention programme during pregnancy on leptin and resistin levels and the factors that influence birth weight, BMI and weight development at one year of life based on data from the ADEBAR and MAMA studies.

## 2. Materials and Methods

### 2.1. Ethical Considerations

Ethical approval for this study was obtained from the Ethics Committees of the German Sport University Cologne (ethics reference number: ADEBAR—108/2014, date of approval 18 December 2014; MAMA—10/07/2012, date of approval 10 July 2012) and the Medical Faculty of the University of Bonn (ethics reference number: 087/15, date of approval 22 April 2014). The study was conducted in accordance with the ethical principles of medical research on humans (Declaration of Helsinki). All study participants provided informed consent, affirming their voluntary participation.

### 2.2. Study Design

The study data were taken from the ADEBAR (Obesity Prevention Through an Exercise- and Nutrition-Based Family Programme) [31] and MAMA study [32]. These studies were (randomised) controlled trials that evaluated the effects of an exercise programme on the course of pregnancy with regard to the offspring. In both programmes, women with singleton pregnancies up to the 13th week of gestation were enrolled. Participants were recruited by hospital—and community—based obstetricians in Cologne, Düsseldorf, and Bonn. Women with pre-existing diabetes mellitus, hypertension, or other comorbidities (e.g., cardiovascular disease, insulin resistance or dyslipidaemia) known to affect foetal growth, using medication for mental health disorders as well as women who were not proficient in German were excluded from the study. Fasting blood samples (food abstinence of 12 h) were taken at three time points: between the 13th and 14th weeks (T1), the 23rd and 24th weeks (T2), and the 35th and 36th weeks (T3) of gestation. Regarding nutritional recommendations the “Nutrition in pregnancy—Practice recommendations of the Network, Healthy Start—Young Family Network” served as the basis for this study [33,34].

#### 2.2.1. Intervention

From the 14th to at least the 36th week of gestation, the women underwent a supervised exercise programme twice a week for at least 60–90 min per session. This programme complied with the international guidelines of physical activity during pregnancy [35], as aerobic and strength-conditioning exercises at a moderate intensity during pregnancy are highly recommended.

Based on dietary recalls, individual dietary counselling took place upon recruitment and during pregnancy. The dietary counselling comprised at least three 90 min sessions and was based on recommendations from the German Health Information Service. Detailed information about the intervention has been published elsewhere [31,32].

#### 2.2.2. Control Group

The women in the control group were also recruited from community-based obstetricians. In case the pregnant woman was not able to participate in the intervention e.g., due to time constraints, she was asked if she would like to participate in the control group. Women in the control group were neither encouraged nor discouraged from exercising. They received routine antenatal care and standard nutrition and activity guidelines from their obstetricians. They also underwent the same physical assessments as the women in the intervention group. To obtain information about physical activity during pregnancy, all pregnant women completed questionnaires at three time points (T1–T3).

### 2.3. Anthropometry

Maternal body weight was measured to the nearest kilogramme using a digital scale (Tanita Corp., Tokyo, Japan), with the participants dressed casually and barefoot; Gestational weight gain was defined as the difference between the self-reported pre-pregnancy weight and the last weight recorded before delivery. The Institute of Medicine recommendations [36] were followed in classifying excessive weight gain. Height was measured using a metal stadiometer, while mid-arm and mid-thigh circumferences were determined using a non-extensible, flexible tape on the right side of the body, with all quantities measured to the nearest 0.1 cm. Skinfold thickness was assessed by an observer using a Harpenden skinfold calliper (John Bull British Indicators Ltd., Harpenden, UK) with a constant pressure of 10 g/mm^2^. The procedure was carefully standardised, and each measurement was made in triplicate on the right side of the body. Four points were measured to the nearest 0.2 mm at the triceps, thigh, subscapularis, and suprailium. The upper arm and thigh fat areas as surrogates for fat mass were estimated on the basis of the circumference of each limb and the mean skinfold thickness using the following formula: UFE = C × (TS/2) and TUA = C^2^/(4π), where UFE is the upper arm/thigh fat area estimate; C is the upper arm/thigh circumference; TS is the triceps skinfold thickness; and TUA is the total upper arm area. The upper arm fat-free area was measured using the following formula: TUA—UFE [37].

The following participant information was retrieved from either medical files or standardised questionnaires: weight before gestation, parity, nationality, level of education, smoking status, and mode of delivery.

The prenatal maternal BMI was calculated using the following formula: body weight (kilogramme)/[body height (metre)]^2^.

Neonatal and children’s data were retrieved from screening examinations after birth and at one year: sex, birth weight, birth length, Apgar score, body weight at one year and body length at one year.

Children’s BMI was calculated as described above. BMI was divided into percentiles according to Voigt et al. [38] (birth weight) or according to Kromeyer-Hauschild et al. [39] (weight at one year). Birth percentile under the 10th percentile was classified as small for gestational age and above the 90th percentile as large for gestational age. Following the guidelines of the Arbeitsgemeinschaft für Adipositas (AGA), a BMI at one year of age under the 10th percentile was classified as underweight, above the 90th percentile as overweight and a BMI above the 97th percentile as obese [40]. In addition, the BMI standard deviation score (SDS) was calculated using the least mean squares (LMS) method for non-normally distributed characteristics [40]:

$${\mathrm{SDS}}_{\mathrm{LMS}}=\frac{{\left[\mathrm{BMI}/M[t]\right]}^{L[t]}-1}{L[t]S[t]}$$

M[t], L[t], and S[t] are parameters for the participant’s age and sex.

### 2.4. Laboratory Parameters

Maternal venous blood samples (7.5 mL serum tube, S-Monovette, Sarstedt, Nümbrecht, Germany) were obtained in a fasting state early in the morning. The samples were centrifuged at 4000 rpm for 10 min at 4 °C in a Hettich MR20 centrifuge (Hettich Lab Technology, Tuttlingen, Germany), and the serum was pipetted into new tubes for storage at −80 °C until evaluation.

Leptin levels were measured using either a direct sandwich enzyme-linked immunosorbent assay (ELISA) kit from MERCK/Millipore, Darmstadt, Germany, a TECAN reader from Nano Quant infinite M200 Pro, Switzerland, or a multiplex immunoassay kit from Bio-Rad Laboratories (Hercules, CA, USA) in accordance with the manufacturer’s instructions.

Resistin levels were measured using a commercially available multiplex immunoassay kit (eBioscience, San Diego, CA, USA or Bio-Rad) in accordance with the manufacturer’s instructions.

### 2.5. Data Analysis and Statistics

Statistical analysis was performed using SPSS (IBM SPSS Statistics for Windows, version 28, IBM Corp., Armonk, NY, USA). Descriptive statistics were applied to present the anthropometric, biomarker, and obstetric data. Mean values and standard deviations were calculated, and statistical significance was indicated by a *p*-value of ≤0.05. All confidence intervals were estimated at the 95% level.

A t-test was performed for two-group comparisons for the metric variables and the χ^2^ and two-sided Fisher tests for the categorical and dichotomous variables. Pearson and Spearman correlations were used to determine significant relationships among the data, and a backward multiple linear regression analysis was employed to assess the individual factors influencing birth weight, BMI at birth, BMI-SDS at birth as well as BMI and BMI-SDS at one year of age.

The initial model for birth weight, BMI and BMI-SDS included the following variables: group (0 = intervention; 1 = control), maternal age (year), pre-pregnancy BMI (kg per meter squared), relative weight gain during pregnancy (percent), estimated upper arm fat area (centimetre squared) at all time points, estimated upper arm fat-free area (centimetre squared) at all time points, resistin level (picogramme per millilitre) at all time points, leptin level (picogramme per millilitre) at all time points, and birth length z-score (except for BMI at birth and BMI-SDS for birth). The initial model for BMI at one year and BMI-SDS at one year included the same variables as described above. In addition, the BMI-SDS at birth was also included in the initial model. All multiple linear regression analyses were tested for multicollinearity and independence of the residuals.

The number of cases in Section 3 (results) may vary. In some cases, the evaluated blood parameters were below the detection limit and therefore excluded from the analysis. In addition, for some pregnant women, the first blood sample could be collected only after the 14th week of pregnancy; their values were not included in the baseline survey. In other cases, the questionnaire was not completely filled out or the measurement of body composition could not be performed or only partially.

## 3. Results

### 3.1. Study Population

A total of 70 women participated in this study: 45 were included in the intervention group and 25 in the control group. Table 1 presents the anthropometric and demographic data of the study sample as well as the differences between the intervention and control groups. The participants in the intervention group were significantly older than those in the control group (*p* = 0.010). No further differences between the groups were found. The study population had a high educational level (83.0%, A-level education), with no differences between the groups. To the best of our knowledge, the participating women had no comorbidities. This was asked several times during the study and was considered an exclusion criterion for participation in the study in many cases (see Section 2.2.). Regarding physical activity, women in the control group were less active than women in the intervention group at all time points (T1: 137.1 ± 132.0 vs. 225.3 ± 94.9 min/week, *p* = 0.006; T2: 90.0 ± 112.1 vs. 239.4 ± 118.7 min/week, *p* ≤ 0.001; T3: 159.8 ± 150.2 vs. 235.4 ± 148.9 min/week, *p* = 0.056).

The obstetric and neonatal data are presented in Table 2. Except for the length at birth, no differences between the groups were found.

### 3.2. Anthropometric Data and Adipokine Levels

The estimated upper arm fat area and total upper arm area were lower in the intervention group than in the control group; however, the difference was not significant (Table 3). The intervention group tended to have more fat-free mass by the end of the intervention than did the control group (*p* = 0.057; Table 3). Nevertheless, there was a positive correlation between the estimated upper arm fat area at T1 and leptin (T1: *p* ≤ 0.001, r = 0.575; T2: *p* = 0.031, r = 0.306; T3: *p* = 0.029, r = 0.312) and resistin levels (T1: *p* ≤ 0.001, r = 0.571; T2: *p* = 0.004, r = 0.395; T3: *p* = 0.038, r = 0.298) at all time points.

The intervention group had significantly lower leptin levels at T1 and T3 than the control group (Table 4). However, the lower leptin level at T3 in the intervention group than in the control group might be attributed to the lower baseline level at T1. Regarding the resistin levels, no differences between the groups were found at any time point.

### 3.3. Birth Weight and Weight Development at One Year

The two groups did not differ significantly with respect to birth weight, BMI at birth, BMI-SDS at birth or birth weight for gestational age (Table 2). Birth weight for gestational age (classification) was positively correlated with leptin at T1 (*p* = 0.027; r = 0.304) and T2 (*p* = 0.025; r = 0.296). Moreover, no further correlations were found between the birth weight, BMI, and BMI-SDS as well as adipokine levels at any time point. However, birth weight for gestational age (classification) significantly correlated with estimated upper arm fat area at T1 (*p* = 0.006; r = 0.373), total upper arm area at T1 (*p* = 0.037; r = 0.290) and T2 (*p* = 0.040; r = 0.276).

A multiple linear regression analysis was performed to evaluate the factors associated with birth weight (Table 5), BMI-SDS at birth (Table 6) and BMI at birth (Supplementary Material Table S1).

In the final model for birth weight, we found that the resistin level at T2 (*β* = −0.259, *p* = 0.015), upper arm fat area at T1 (*β* = 1.121, *p* ≤ 0.001) and T3 (*β* = −0.682, *p* ≤ 0.001), and birth length z-score (*β* = 0.446, *p* ≤ 0.001) were significantly associated with the birth weight. These variables explained 62.2% of the variance. Further details are presented in Table 5. In order to consider birth weight in relation to birth length and gender, another linear regression was calculated with BMI-SDS as the outcome variable (Table 6). In the final model, the influencing variables resistin level at T2 and upper arm fat area at T1 as well as T3 are also confirmed. The variables explained 33.6% of the variance.

In terms of weight development at one year of age, children from the two groups did not differ significantly in weight, BMI or BMI-SDS (Table 7). BMI classification at one year of life was positively correlated with resistin level at T3 (*p* = 0.026; r = 0.306). Moreover, no further correlations were found between the body weight, BMI, BMI-SDS and adipokine levels at any time point.

A multiple linear regression analysis was performed to evaluate the factors associated with BMI at one year of age (Supplementary Material Table S2) and BMI-SDS at one year of age (Table 8). In the final model for BMI-SDS at one year of age, we found that the resistin level at T1 (*β* = −0.415, *p* = 0.079) and T2 (*β* = 0.572, *p* = 0.017) and upper arm fat-free area at T3 (*β* = 0.296, *p* = 0.069) were significantly associated with the BMI-SDS. These variables explained 13.3% of the variance (Table 8). The multiple linear regression analysis with BMI as the outcome variable also confirms the final and influencing variables (Supplementary Material Table S2).

## 4. Discussion

To our knowledge, the current study is one of the first to examine the influence of an exercise and nutrition programme during pregnancy on maternal leptin and resistin levels and birth weight as well as weight development up to one year of life. Independent of the intervention, the resistin and leptin levels correlated with the maternal fat area as surrogates for fat mass at the beginning of pregnancy; fat-free area/mass tended to increase at the end of the intervention in the intervention group and decreased in the control group. The intervention only had a slight influence on the laboratory parameters. The leptin level was significantly higher in the control group at T3 but was already lower in the intervention group before the start of the intervention. The birth weight as well as the BMI-SDS at birth were influenced by the birth length, fat area/fat mass at T1 and T3, and resistin level at T2. The child’s body weight at one year also seems to be influenced by resistin at T1 and T2 as well as by the mother’s body composition during pregnancy.

Body weight naturally increases during pregnancy, often accompanied by an increase in the fat area/fat mass. Nien et al. identified higher resistin levels at the end of pregnancy than at the first trimester, justifying this observation with a possible connection between the fat area/fat mass, the resistin level, and insulin resistance during pregnancy [41]. Although we could not demonstrate a significant increase in the resistin or leptin levels during pregnancy, our results showed a clear association between the fat area/fat mass at the beginning of pregnancy and adipokine levels at all three time points. In future multimodal interventions, the body composition should possibly be better considered and included in the evaluation. An even more targeted exercise programme could possibly increase muscle mass more significantly and influence fat area/mass, thus indirectly influencing adipokine levels.

The influence of physical activity on leptin levels has already been investigated by Clapp et al., who demonstrated an almost linear increase in leptin levels as pregnancy progressed; however, this increase was reduced by exercise at all time points [15]. Additionally, Ning et al. revealed that the mean leptin levels were lower in women with the highest levels of physical activity (>12.8 h/week) and energy expenditure (>70.4 MET h/week) than in inactive women during early pregnancy (average of 12–13 weeks of gestation) [26]. Previous studies have also found a negative correlation between maternal leptin levels and sports exercise performance [25]. With respect to offspring, we also found a positive association between maternal leptin levels at the end of pregnancy and changes in infant BMI-SDS over the first year of life [42].

The results on resistin and their clinical relevance remain unclear, even though the resistin level at T2 and the fat area explained 62.2% of the variance in the regression analysis with respect to the birth weight. Taking body weight in relation to body length and gender into account, the variables remained the same. Moreover, resistin at T1 and T2 as well as maternal free-fat area/mass at T3 explained 13.3% of the variance with respect to BMI-SDS at one year of life.

Early studies have suggested an inverse association between maternal resistin and foetal LDL-cholesterol levels, indicating that the status of maternal adiposity may play an active role in the regulation of the foetal lipid profile and, consequently, foetal programming [43,44]. In our previous cross-sectional study among 110 pregnant women, we analysed factors influencing resistin levels using multiple linear regression and found that the levels were not associated with any of the investigated variables (maternal age, pre-pregnancy BMI, physical activity from the first to the third trimester, parameters of body composition, and healthy eating index) [45]. In terms of the association of nutritional status with resistin levels, Fargnoli et al. indicated that close adherence to a healthy dietary pattern led to lower levels of resistin and other biomarkers of inflammation in a non-pregnant cohort [46]. In contrast, Millar et al. were unable to find any changes in resistin levels in middle-aged individuals resulting from maintaining a healthy diet [47]. However, the data on the correlation between resistin levels and birth weight and the influence on metabolic dysregulation are heterogeneous. Nevertheless, resistin levels and thus the metabo-inflammatory profile in non-pregnant women is positively influenced by physical activity. For example, in their pilot study among 12 women with an average age of 35 years, Amorim et al. found significant reductions in not only resistin (68.4%), leptin (35.0%), TNF-α (57.0%), and CRP (84.7%) levels but also markers of body composition, such as body weight (5.6%) or percentage body fat (10.9%), after a 1-month exercise programme (exergame for 1 h/day) [48].

Although data about resistin are limited in the context of pregnancy, the results underline the relevance of a healthy lifestyle during pregnancy, considering the known associations with the development of insulin resistance. For example, Houshmand-Oeregaard et al. found no significant changes in plasma leptin, resistin, and adiponectin levels in their study of the offspring of mothers with and without GDM [49]. However, analyses of DNA methylation and gene expression have shown significantly increased adiponectin DNA methylation and decreased adiponectin and resistin gene expression in the subcutaneous adipose tissue of the offspring of mothers with GDM. Jönsson et al. evaluated the umbilical cord blood of 208 offspring whose mothers were included in either a lifestyle intervention group (diet and exercise) or a control group (no intervention) [50]. The intervention affected the DNA methylation at 397 sites, and the changes could be assigned to genes for adipose tissue development or responses to fatty acids, among other factors. Therefore, epigenetic analyses could provide new insights in future studies. One can only speculate to what extent this can be transferred to the context of pregnancy; birth weight; and thus, in the long term, offspring.

### Limitations

The present study has several limitations. The study population was involved in two studies; however, the content and implementation of the studies were the same. The exercise and nutrition programme and the examinations were conducted by trained sports scientists/dieticians from the German Sport University Cologne under the same supervision. The participants were recruited from the region around Cologne-Bonn and Düsseldorf only. Therefore, our sample may not be representative of the general population. Moreover, the increased occurrence of specific subpopulations arising from, for example, an increased interest in study participation among health-conscious individuals with good fitness levels, cannot be ruled out.

The blood parameters were all measured via ELISA or multiplex ELISA, although not with the same test kits. An internal control for deviating mean values revealed no significant differences. In addition, the blood samples were taken at three different time points during pregnancy (14th, 24th, and 36th weeks of gestation). Comparisons with other studies are therefore difficult in some cases owing to the use of different time points. Furthermore, it is critical to note that the plasma levels of leptin and resistin reveal no insight into the effect on the end organ. Effects with regard to receptor function are possible; however, this would have to be tested in animal experiments.

The assessment of fat area/fat mass and fat-free area/fat-free mass was based on the circumference of each limb and the mean skinfold thickness. Although the measurement methods were standardised and performed only by trained staff, they have some limitations. Performing a bioimpedance analysis would notably have yielded more accurate results but is not allowed during pregnancy. Moreover, the determination of fitness as a possible surrogate parameter would have been useful.

## 5. Conclusions

A multimodal lifestyle intervention during pregnancy may partially impact maternal body composition and the levels of selected maternal adipokines. Even though resistin was not directly affected by our multimodal lifestyle intervention, it shows that, for example, the intervention group tended to increase fat-free mass at T3 compared to the control group. This, as well as resistin, in turn, seems to have an influence on BMI-SDS in the first year of life.

The heterogeneous results obtained herein reflect previous literature to a certain extent. However, they also show that lifestyle interventions in pregnancy are important in promoting maternal and child health. Therefore, further randomised controlled trials with larger study populations and translational studies are warranted to confirm our results and clarify the underlying mechanisms.

## Figures and Tables

**Table 1:** Anthropometric and demographic data of the total sample population and each group at baseline.

Parameter	Total Population Mean ± SD/%	Intervention Group Mean ± SD/%	Control Group Mean ± SD/%	*p*-Value
Age (year)	32.7 ± 4.7 (n = 70)	33.7 ± 4.9 (n = 45)	30.8 ± 3.9 (n = 25)	0.010 *
Height (m)	1.67 ± 0.05 (n = 69)	1.67 ± 0.06 (n = 44)	1.66 ± 0.05 (n = 25)	0.377
Pre-pregnancy weight (kg)	75.1 ± 19.5 (n = 69)	75.5 ± 18.6 (n = 44)	74.4 ± 21.4 (n = 25)	0.821
Pre-pregnancy BMI (kg/m^2^)	26.3 ± 6.5 (n = 69)	26.5 ± 6.2 (n = 44)	26.1 ± 7.1 (n = 25)	0.833
Pre-pregnancy BMI classification				
Normal weight	59.4 % (n = 41)	61.4 % (n = 27)	56.0 % (n = 14)	0.214
Overweight	14.5 % (n = 10)	9.1 % (n = 4)	24.0 % (n = 6)	
Obese	26.1 % (n = 18)	29.5 % (n = 13)	20.0 % (n = 5)	
Nationality (German)	90.0 % (n = 63)	88.9 % (n = 40)	92.0 % (n = 23)	0.494
Primipara	72.0 % (n = 53)	76.1 % (n = 35)	65.5 % (n = 19)	0.232
Current smoker (yes)	2.9 % (n = 2)	2.2 % (n = 1)	4.0 % (n = 1)	0.590

The data are presented as means ± standard deviations or % (n). Abbreviations: BMI = body mass index; * unpaired *t*-test.

**Table 2:** Obstetric and neonatal data of the total sample population and each group.

Parameter	Total Population Mean ± SD/%	Intervention Group Mean ± SD/%	Control Group Mean ± SD/%	*p*-Value
**Obstetric data**				
Gestational age at delivery (weeks)	39.7 ± 1.3 (n = 62)	40.0 ± 1.2 (n = 37)	39.3 ± 1.5 (n = 25)	0.073
Weight gain during pregnancy (kg)	14.9 ± 6.6 (n = 60)	15.7 ± 5.1 (n = 37)	13.5 ± 8.5 (n = 23)	0.282
Weight gain classification (IOM)				0.173
Lower than the recommendation	18.6% (n = 11)	16.2% (n = 6)	22.7% (n = 5)	
Within the recommendation	33.9% (n = 20)	27.0% (n = 10)	45.5% (n = 10)	
Higher than the recommendation	47.5% (n = 28)	56.8% (n = 21)	31.8% (n = 7)	
Mode of delivery				0.851
Normal vaginal delivery	27.9% (n = 17)	27.8% (n= 10)	28.0% (n = 7)	
Instrumental vaginal delivery	4.6% (n = 3)	5.6% (n = 2)	4.0% (n = 1)	
Elective caesarean section	16.4% (n = 10)	19.4% (n = 7)	12.0% (n = 3)	
Emergency caesarean section	50.8% (n = 31)	47.2% (n = 17)	56.0% (n = 14)	
**Neonatal data**				
Female sex	57.6% (n = 38)	62.5% (n = 25)	50.0% (n = 13)	0.227
Birth weight (g)	3495.1 ± 464.6 (n = 65)	3558.1 ± 445.6 (n = 39)	3400.6 ± 485.1 (n = 26)	0.191
Birth length (cm)	51.8 ± 2.5 (n = 65)	52.3 ± 2.3 (n = 39)	51.0 ± 2.6 (n = 26)	0.048 *
BMI (kg/m^2^)	13.0 ± 1.2 (n = 65)	13.0 ± 1.2 (n = 39)	13.0 ± 1.3 (n = 26)	0.877
BMI-SDS	0.3 ± 1.0 (n = 65)	0.3 ± 1.0 (n = 39)	0.3 ± (n = 26)	0.884
Birth weight classification according to gestational age				0.264
Small for gestational age (<10. Percentile)	3.2% (n = 2)	5.4% (n = 2)	0% (n = 0)	
Appropriate for gestational age (10.–90. Percentile)	82.3% (n = 51)	81.1% (n = 30)	84.0% (n = 21)	
Large for gestational age (>90. Percentile)	11.5% (n = 9)	13.5% (n = 5)	16.0% (n = 4)	
5 min Apgar score of >7	95.1% (n = 58)	94.6% (n= 35)	95.8% (n = 23)	0.660
10 min Apgar score of ≥9	100% (n = 61)	100% (n = 37)	100% (n = 24)	-

The data are presented as means ± standard deviations or% (n). Abbreviations: IOM = Institute of Medicine; * unpaired *t*-test.

**Table 3:** Estimated maternal fat mass and fat-free mass at three time points during pregnancy in the total sample population and each group.

Parameter	Total Population Mean ± SD	Intervention Group Mean ± SD	Control Group Mean ± SD	*p*-Value
T1 Total upper arm area (cm^2^)	74.6 ± 29.2 (n = 62)	73.7 ± 27.1 (n = 38)	76.1 ± 32.7 (n = 24)	0.763
T2 Total upper arm area (cm^2^)	72.5 ± 24.5 (n = 62)	73.1 ± 25.0 (n = 41)	71.5 ± 24.5 (n = 21)	0.812
T3 Total upper arm area (cm^2^)	71.6 ± 24.1 (n = 58)	72.3 ± 25.6 (n = 37)	70.9 ± 21.8 (n = 21)	0.831
T1 Upper arm fat area (cm^2^)	32.2 ± 18.4 (n = 62)	29.6 ± 14.6 (n = 38)	36.5 ± 23.1 (n = 24)	0.153
T2 Upper arm fat area (cm^2^)	29.3 ± 15.0 (n = 62)	28.6 ± 13.4 (n = 41)	30.6 ± 18.3 (n = 21)	0.661
T3 Upper arm fat area (cm^2^)	29.2 ± 19.3 (n =59)	28.0 ± 18.2 (n = 38)	31.4 ± 21.5 (n = 21)	0.538
T1 Upper arm fat-free area (cm^2^)	42.4 ± 15.8 (n = 62)	44.1 ± 17.4 (n = 38)	39.6 ± 17.7 (n = 24)	0.248
T2 Upper arm fat-free area (cm^2^)	43.2 ± 16.1 (n = 62)	44.4 ± 17.6 (n = 41)	40.9 ± 12.6 (n = 21)	0.369
T3 Upper arm fat-free area (cm^2^)	44.7 ± 17.6 (n = 59)	47.6 ± 19.4 (n = 38)	39.5 ± 12.6 (n = 21)	0.057

The data are presented as means ± standard deviations (n). T1, T2, and T3 represent around 14, 24, and 36 weeks of gestation, respectively.

**Table 4:** Maternal leptin and resistin levels at three time points during pregnancy in the total sample population and each group.

Parameter	Total Population Mean ± SD	Intervention Group Mean ± SD	Control Group Mean ± SD	*p*-Value
T1 Leptin level (pg/mL)	18,071.2 ± 12,694.7 (n = 56)	13,854.3 ± 8133.4 (n = 36)	25,661.7 ± 15,821.0 (n = 20)	≤0.001 *
T2 Leptin level (pg/mL)	22,534.7 ± 18,701.3 (n = 60)	19,916.1 ± 13,955.1 (n = 39)	27,397.9 ± 24,970.0 (n = 21)	0.141
T3 Leptin level (pg/mL)	20,265.2 ± 13,698.4 (n = 58)	17,042.0 ± 10,542.7 (n = 38)	26,389.3 ± 16,918.5 | (n = 20)	0.033 *
T1 Resistin level (pg/mL)	10,803.5 ± 4435.9 (n = 56)	10,766.2 ± 3466.3 (n = 36)	10,870.5 ± 5900.9 (n = 20)	0.943
T2 Resistin level (pg/mL)	11,428.2 ± 5549.9 (n = 60)	11,734.7 ± 3912.4 (n = 39)	10,859.1 ± 7826.6 (n = 21)	0.564
T3 Resistin level (pg/mL)	20,265.2 ± 13,698.4 (n =58)	10,278.9 ± 2676.2 (n = 38)	8921.9 ± 3678.9 (n = 20)	0.155

The data are presented as means ± standard deviations (n). T1, T2, and T3 represent around 14, 24, and 36 weeks of gestation, respectively; * unpaired *t*-test.

**Table 5:** Backward multiple linear regression analysis with birth weight as the outcome variable (first ^§^ and final models).

Model ^§^		*β-*Coefficient	*p*-Value	R^2^
1	Maternal age [year]	0.067	0.639	0.569
	Group	−0.025	0.885	
	Realtive weight gain during pregnancy [%]	−0.019	0.907	
	Upper arm fat area at T1 [cm^2^]	1.117	≤0.001	
	Upper arm fat area at T3 [cm^2^]	−0.892	0.008	
	Leptin level at T1 [pg/mL]	0.232	0.334	
	Leptin level at T2 [pg/mL]	−0.072	0.664	
	Leptin level at T3 [pg/mL]	0.078	0.727	
	Resistin level at T1 [pg/mL]	0.200	0.301	
	Resistin level at T2 [pg/mL]	−0.454	0.034	
	Resistin level at T3 [pg/mL]	0.083	0.561	
	Birth length z-score	0.445	≤0.001	
9	Upper arm fat area at T1 [cm^2^]	1.121	≤0.001	0.622
	Upper arm fat area at T3 [cm^2^]	−0.682	≤0.001	
	Resistin level at T2 [pg/mL]	−0.259	0.015	
	Birth length z-score	0.418	≤0.001	

^§^ Due to multicollinearity “Pre-pregnancy BMI, Upper arm fat area at T2 and Upper arm fat-free area at T1/T2/T3” was excluded from analysis. Abbreviations: BMI = body mass index; T1, T2, and T3 represent around 14, 24, and 36 weeks of gestation, respectively.

**Table 6:** Backward multiple linear regression analysis with BMI-SDS at birth as the outcome variable (first ^§^ and final models).

Model ^§^		*β-*Coefficient	*p*-Value	R^2^
1	Maternal age [year]	−0.092	0.620	0.243
	Group	−0.099	0.656	
	Pre-pregnancy BMI [kg/m^2^]	−0.258	0.492	
	Realtive weight gain during pregnancy [%]	−0.084	0.715	
	Upper arm fat area at T1 [cm^2^]	1.158	0.009	
	Upper arm fat area at T3 [cm^2^]	−0.677	0.100	
	Leptin level at T1 [pg/mL]	0.129	0.687	
	Leptin level at T2 [pg/mL]	−0.221	0.324	
	Leptin level at T3 [pg/mL]	0.304	0.258	
	Resistin level at T1 [pg/mL]	0.015	0.954	
	Resistin level at T2 [pg/mL]	−0.453	0.103	
	Resistin level at T3 [pg/mL]	0.166	0.386	
10	Upper arm fat area at T1 [cm^2^]	1.008	≤0.001	0.336
	Upper arm fat area at T3 [cm^2^]	−0.579	0.023	
	Resistin level at T2 [pg/mL]	−0.319	0.021	

^§^ Due to multicollinearity “Upper arm fat area at T2 and Upper arm fat-free area at T1/T2/T3” was excluded from analysis. Abbreviations: BMI = body mass index; T1, T2, and T3 represent around 14, 24, and 36 weeks of gestation, respectively.

**Table 7:** Children’s anthropometric data of the total sample population and each group one year after the intervention.

Parameter	Total Population Mean ± SD/%	Intervention Group Mean ± SD/%	Control Group Mean ± SD/%	*p*-Value
Age (year)	1.0 ± 0.1 (n = 60)	1.0 ± 0.2 (n = 35)	1.0 ± 0.05 (n = 25)	0.100
Height (cm)	75.5 ± 2.8 (n = 60)	75.8 ± 2.5 (n = 35)	75.1 ± 3.3 (n = 25)	0.386
Body weight (Kg)	9.5 ± 1.1 (n = 60)	9.6 ± 1.1 (n = 35)	9.4 ± 1.1 (n = 25)	0.478
BMI (Kg/m^2^)	16.7 ± 1.6 (n = 60)	16.7 ± 1.5 (n = 35)	16.7 ± 1.8 (n = 25)	0.958
BMI-SDS	−0.03 ± 1.2 (n = 60)	0.02 ± 1.0 (n = 35)	−0.09 ± 1.5 (n = 25)	0.726
BMI classification				0.388
Underweight (<10.P)	11.7 (n = 7)	8.6% (n = 3)	16.0% (n = 4)	
Normal weight (11–90.P)	76.7% (n = 46)	82.9% (n = 29)	68.0% (n = 17)	
Overweight (91–97.P)	6.7% (n = 4)	2.9% (n = 1)	12.0% (n = 3)	
Obese (>97.P)	5.0% (n = 3)	5.7% (n = 2)	4.0% (n = 1)	

The data are presented as means ± standard deviations (n). Abbreviations: BMI = body mass index; .P = percentile.

**Table 8:** Backward multiple linear regression analysis with BMI-SDS at one year of age as the outcome variable (first ^§^ and final models).

Model ^§^		*β-*Coefficient	*p*-Value	R^2^
1	Maternal age [year]	−0.024	0.597	−0.052
	Group	−0.214	0.434	
	Realtive weight gain during pregnancy [%]	0.030	0.323	
	Upper arm fat-free area at T1 [cm^2^]	−0.141	0.224	
	Upper arm fat-free area at T2 [cm^2^]	−0.130	0.261	
	Upper arm fat-free area at T3 [cm^2^]	0.452	0.213	
	Leptin level at T1 [pg/mL]	−0.007	0.247	
	Leptin level at T2 [pg/mL]	0.124	0.403	
	Leptin level at T3 [pg/mL]	−0.007	0.204	
	Resistin level at T1 [pg/mL]	−0.459	0.300	
	Resistin level at T2 [pg/mL]	0.705	0.209	
	Resistin level at T3 [pg/mL]	−0.151	0.465	
	BMI-SDS at birth	0.072	0.718	
11	Upper arm fat-free area at T3 [cm^2^]	0.296	0.069	0.133
	Resistin level at T1 [pg/mL]	−0.415	0.079	
	Resistin level at T2 [pg/mL]	0.572	0.017	

^§^ Due to multicollinearity “Upper arm fat area at T1/T2/T3” was excluded from analysis. Abbreviations: BMI = body mass index; T1, T2, and T3 represent around 14, 24, and 36 weeks of gestation, respectively.

## Data Availability

Not applicable.

## Supplementary Materials

The following supporting information can be downloaded at: https://www.mdpi.com/article/10.3390/biomedicines11020447/s1, Table S1: Backward multiple linear regression analysis with BMI at birth as the outcome variable (first and final models); Table S2: Backward multiple linear regression analysis with BMI at one year of age as the outcome variable (first and final models).
