# Supplementary material for: Effect of Lifestyle Interventions during Pregnancy on Maternal Leptin, Resistin and Offspring Weight at Birth and One Year of Life
Source: Biomedicines. 2023 Feb 3;11(2):447. doi: 10.3390/biomedicines11020447 (PMC9953512; doi:10.3390/biomedicines11020447)
Supplement: Supplementary file 1 [file biomedicines-11-00447-s001.zip › biomedicines-2177781-supplementary.pdf]

Supplemental Material

Table S1. Backward multiple linear regression analysis with BMI at birth as the outcome variable (first[§] and final models).

Model[§]		β-coefficient	P-value	R^2
1	Maternal age [year]	-0.062	0.741	0.229
	Group	-0.100	0.655	
	Pre-pregnancy BMI [kg/m^2]	-0.256	0.500	
	Realtive weight gain during pregnancy [%]	-0.095	0.683	
	Upper arm fat area at T1 [cm^2]	1.163	0.009	
	Upper arm fat area at T3 [cm^2]	-0.721	0.083	
	Leptin level at T1 [pg/mL]	0.144	0.655	
	Leptin level at T2 [pg/mL]	-0.203	0.368	
	Leptin level at T3 [pg/mL]	0.302	0.264	
	Resistin level at T1 [pg/mL]	0.035	0.894	
	Resistin level at T2 [pg/mL]	-0.456	0.104	
	Resistin level at T3 [pg/mL]	0.127	0.508	
10	Upper arm fat area at T1 [cm^2]	1.016	≤0.001	0.332
	Upper arm fat area at T3 [cm^2]	-0.601	0.019	
	Resistin level at T2 [pg/mL]	-0.315	0.023	

[§] Due to multicollinearity "Upper arm fat area at T2 and Upper arm fat-free area at T1/T2/T3" was excluded from analysis. Abbreviations: BMI = body mass index; T1, T2, and T3 represent around 14, 24, and 36 weeks of gestation, respectively

Table S2. Backward multiple linear regression analysis with BMI at one year of age as the outcome variable (first§ and final models).

**Model§**		***β*-coefficient**	***P*-value**	**R^2**
1	Maternal age [year]	0.113	0.590	-0.006
	Group	-0.082	0.739	
	Realtive weight gain during pregnancy [%]	-0.025	0.929	
	Upper arm fat-free area at T1 [cm²]	-0.201	0.558	
	Upper arm fat-free area at T2 [cm²]	-0.194	0.542	
	Upper arm fat-free area at T3 [cm²]	0.609	0.092	
	Leptin level at T1 [pg/mL]	-0.071	0.828	
	Leptin level at T2 [pg/mL]	0.102	0.691	
	Leptin level at T3 [pg/mL]	0.041	0.909	
	Resistin level at T1 [pg/mL]	-0.409	0.175	
	Resistin level at T2 [pg/mL]	0.721	0.050	
	Resistin level at T3 [pg/mL]	-0.175	0.465	
	BMI-SDS at birth	0.098	0.612	
10	Upper arm fat-free area at T1 [cm²]	-0.323	0.094	0.198
	Upper arm fat-free area at T3 [cm²]	0.534	0.011	
	Resistin level at T1 [pg/mL]	-0.382	0.097	
	Resistin level at T2 [pg/mL]	0.585	0.012	

§ Due to multicollinearity "Pre-pregnancy BMI and Upper arm fat area at T1/T2/T3" was excluded from analysis. Abbreviations: BMI = body mass index; T1, T2, and T3 represent around 14, 24, and 36 weeks of gestation, respectively
